# The Simplified BrainTower and Pipe Cleaners: Model Building as a Learning Tool in Neuroscience

**DOI:** 10.59390/001c.153902

**Published:** 2025-12-31

**Authors:** Andrew J Doherty, Joanna L Howarth

**Affiliations:** 1 School of Physiology, Pharmacology and Neuroscience University of Bristol; 2 Bristol Medical School University of Bristol

**Keywords:** model, neuroanatomy, ascending tracts, descending tracts, anterolateral, spinothalamic, corticospinal, dorsal column, Simplified BrainTower

## Abstract

Models have a long history of use in helping students to understand the three-dimensional organisation of biological structures. This is particularly true of neuroanatomical teaching where it is often difficult to distinguish grey and white matter in the fixed prosections often used in dissection rooms. A model system designed to help medical students understand the anatomy of the ascending and descending white matter tracts through the brain and spinal cord had the opposite effect: it made the task of learning these tracts more difficult. The model was complex and difficult for students to engage with. The aim of this study was to introduce a simplified version of this modelling system that was interactive and intuitive, to enable students to build a model in a single workshop session and to assess how the modeling activity impacted on students’ learning. Students were asked to build a model of either the anterolateral/spinothalamic tracts (ALS), the dorsal column/medial lemniscus tract (DCML) or the corticospinal tracts (CST) using pipe-cleaners to represent the route of the pathway, including the number of neurons. We found that students were easily able to build a representation of one of these tracts and that they could answer questions on the structure and function of the tract they built significantly better than similar questions related to the other tracts. This enhancement in knowledge was maintained through to end of unit exams eleven weeks after the modelling activity, demonstrating that actively building a model contributes to deep rather than superficial learning.

Models have a long history of use in helping students understand the 3-dimensional (3D) organization of biological structures (Narang et al., 2021). Visualization of 3-dimensional structures from 2-dimensional images is a complex process, requiring the ability to construct a mental model that can be rotated in different directions. This kind of spatial ability is correlated with the successful use of 3D computer models (Huk, 2006) and it has been shown that the use of both physical and computer models enhances students’ understanding of 3D structures. For instance, the disassembly of physical models has been shown to greatly enhance learning of the structure of DNA and its component parts (Srivastava, 2016). Similar results have been found using peptide models for the understanding of protein structures and digital chemical models for the understanding of 3D molecular structure (Barak & Hussein-Farraj, 2012). This latter study showed that students are more engaged and have better outcomes if they can manipulate the computer model themselves.

There have been many attempts to provide manipulatable, bespoke and open source computer-based 3D resources for teaching aspects of human anatomy (Allen et al., 2016; Brenton et al., 2007; Petersson et al., 2009). While these systems have advantages in that students can view these models from multiple devices, all have suffered from requiring separate plug-ins for each internet browser that allows users to manipulate the models, many of which are Java™ based and blocked by current digital security systems. The advent of HTML5 and WebGL technology has meant that any modern browser can display a manipulatable 3D model that is hosted on a suitable website such as Sketchfab (www.sketchfab.com). However, few such resources are currently available at a reasonable cost. In addition, the use of models which can be held and manipulated by hand has been shown to be better at engaging students and improving assessment outcomes than digital models (Khot et al., 2013).

Anatomical teaching is an area that lends itself to the use of models and neuroanatomy has long been seen as a particularly difficult topic for students (Flanagan et al., 2007; Kramer & Soley, 2002; Schon et al., 2002). This has been particularly true for teaching the anatomy of white matter tracts as the individual pathways cannot be seen in the fixed prosections that are often used in dissection classes (Arnts et al., 2014). However, the ascending sensory and descending motor tracts are very well described and so are well-suited to a discussion of the functional anatomy of the central nervous system. They clearly demonstrate that there are regions of the cortex that are devoted to specific functional roles and that knowing the anatomy is a key part of understanding their functions. The defined anatomy of these tracts also makes them well-suited to the use of models. In addition, in our experience students find the ascending and descending tracts challenging to learn, in particular the points of decussation and the location of synapses. When asked which topics they wish to review in revision sessions, students will often request additional time spent on these tracts.

To try and counter these difficulties, a model system for teaching the ascending and descending tracts in the brain and spinal cord was developed here at Bristol University - the BrainTower^®^ (Greene, 2007, 2008, 2009). This system consisted of plastic plates that represent sections through the brain and spinal cord at different levels relating to axial MRI images, with holes to represent a wide range of ascending and descending pathways (see Fig 1). The fiber bundles that make up the pathways were represented by colored plastic wires which were fed through the holes and linked to each other by shaped plastic components to represent the different nuclei and cortical target regions. It was originally designed for students to be able to build the models themselves and so gain an understanding of the functions of particular nerves and nuclei and to appreciate the flow of information through the tracts. However, each model consisted of components that were very small and easily lost. It was difficult for students themselves to build the models in a reasonable time frame and was thus ineffective as an interactive teaching aid. Indeed, when asked, 77% of undergraduate medical students in their 2^nd^ year of study disagreed with the proposition that the model aided their understanding of neuroanatomy while 73% agreed that it actually made learning neuroanatomy *more* difficult than it was already perceived to be (Davies et al., 2014).

In this light, while the concept of the BrainTower^®^ is sound, it requires modification to make it suitable for first year undergraduate students studying the anatomy of white matter tracts in the CNS. Our goal was therefore to create an improved active learning tool, allowing students to physically interact with the model and so gain an understanding of the structure of the ascending and descending tracts. We implemented a simplified version of the BrainTower^®^ with fewer tracts represented than the original, which students used to create models of one of three spinal cord tracts. Pipe cleaners were used to represent different neurons that make up each pathway. Within a teaching session, and with the support of staff, students were encouraged to discuss the anatomical features that were required to be represented. In subsequent tests, individual students answered questions about the model they had built more effectively than questions about the models they had not built. This effect lasted 11 weeks after the modeling session, demonstrating that active modeling and discussion instilled long-lasting learning. Finally, the modeling activity with the Simplified BrainTower was seen as useful by students.

## MATERIALS AND METHODS

### Model design and construction

**Figure 1.**
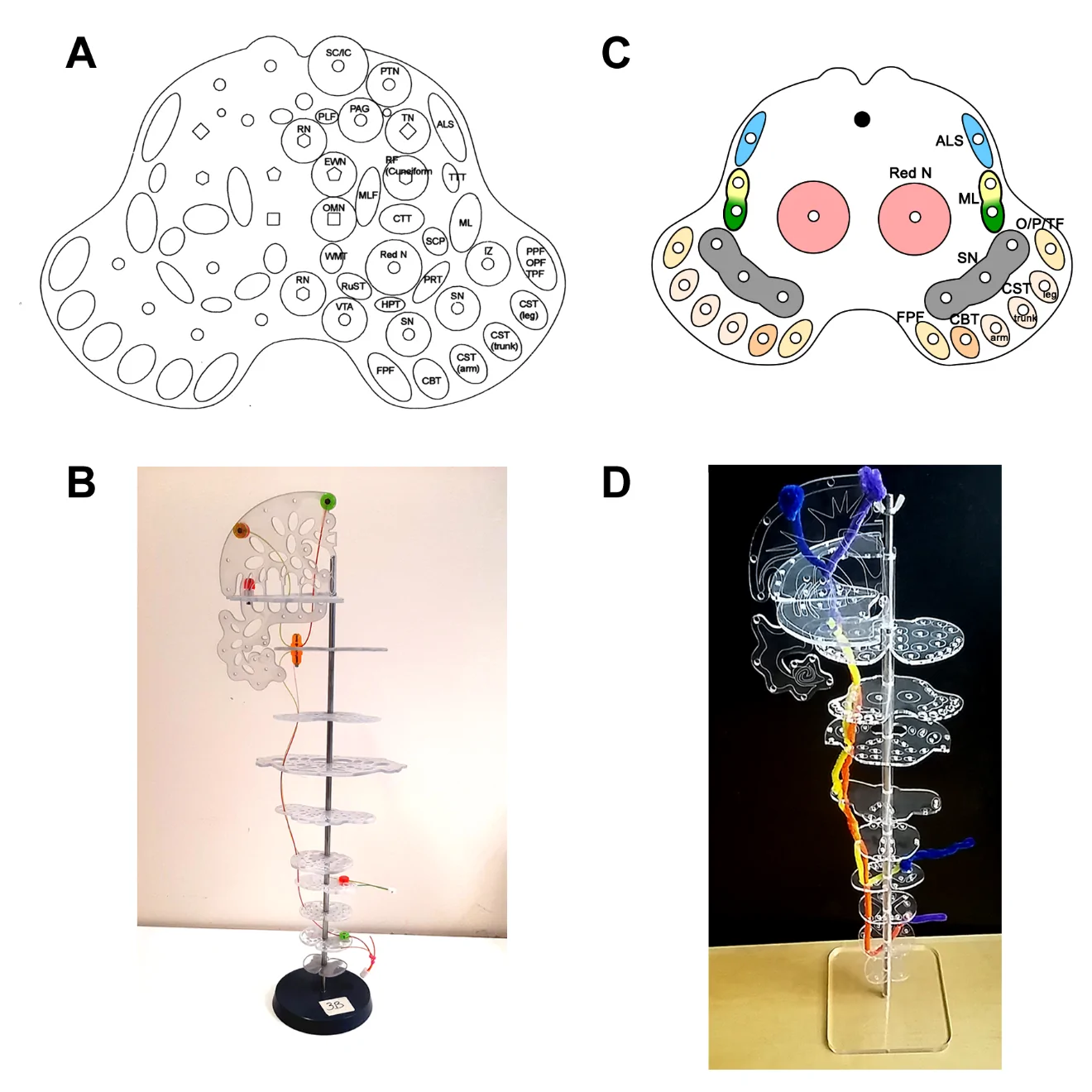
Modifications to the BrainTower® modeling system. Panels A and B – show the original design of the modeling system. Panel A shows the midbrain plate, which contains 30 separate named elements. Panel B shows a completed BrainTower® model of the anterolateral/spinothalamic tract with the extra elements incorporated to show the positions of nuclei and synapses. Panels C and D show the modified modeling system. Panel C shows the modified version of the midbrain plate. It contains fewer named elements than in the original design (11 vs 30) and is scaled to 75% of the original size. The two elements of the medial lemniscus (ML) have been depicted in the new version. Elements represented are: ALS – anterolateral/spinothalamic tract, Red N – red nucleus, ML – medial lemniscus, SN - substantia nigra, FPF – frontopolar fibers, CBT – corticobulbar tract, CST – corticospinal tract, O/P/TF – occipital, parietal, temporal fibers, arm, trunk and leg represent the regions of the crus cerebri containing CST fibers devoted to control of those parts of the body. Panel D shows a model of the anterolateral/spinothalamic tract (ALS) equivalent to that shown in panel B built by one group of students. Colored pipe cleaners represent the neurons in the pathway. In this case, there are three neurons in the pathway, 1^st^, 2^nd^ and 3^rd^ order sensory neurons, which are represented by blue/yellow/blue and purple/orange/purple pipe cleaner combinations. Decussation is depicted at the entry point to the spinal cord for both the arms (blue pipe cleaner) and legs (purple pipe cleaner).

The Simplified BrainTower system was based on an original design produced in Bristol, UK, as an interactive tool for teaching 2^nd^ year undergraduate medical students (Greene, 2007, 2008, 2009). Following poor initial student feedback, several modifications were made to make it more suitable for students at an earlier point in their schooling: their first semester. (Fig 1).

The original design consisted of 11 clear plastic plates (4mm thick) representing sections of the spinal cord (sacral, lumbar, thoracic and cervical sections), the brainstem (medulla, pons and midbrain), thalamus and cerebrum (horizontal and coronal sections). Each plate was etched with a frosted pattern to represent internal gray matter structures and pierced with numerous holes to represent the different white matter tracts. The designs were copied to Autodesk CAD 2016, where they were modified and sent to an internal workshop for manufacture.

Our first modification focused on reducing the overall size and complexity of the model. The plates, including piercings, were laser cut from 2mm acrylic sheets and scaled to 75% of original size. The outlines of subcortical gray matter structures were etched on the surface rather than frosted as in the original design. The plates thus looked similar to sections seen on histological slides or fixed prosections students had seen previously in neuroanatomy classes. While the number of plates from the original design was retained, the number of tracts represented was greatly reduced. For example, in the midbrain plate, the number of specific tracts represented was reduced from 30 to 11 (Fig 1A, B). However, we also included both the gracile and cuneate derived tracts of the medial lemniscus, a detail not included in the original model but often taught in lectures and featured in textbooks and online resources.

We also modified how the model was to be built by the students. In the original design, each plate was clamped between short metal rods that had to be screwed into the rod below. This is a time-consuming method of construction, especially if the model needs to be taken apart to correct an error. We thus re-designed this to have a single central steel rod fixed on a 150 x 150 mm base plate. Clear acrylic tubes were then used to separate the plates representing the anatomical sections with the whole tower secured by a butterfly nut at the top (Fig 1C, D).

The final modification was in how the tracts themselves were represented. In the original model, this was achieved by using plastic wires and molded-plastic components to represent synapses and nuclei. This was very difficult for students to build as it required several tools and many small screws that were easily lost. We thus used colored pipe cleaners that could be fed through the holes and joined together to represent synapses. This is a much more intuitive method of construction with students instantly recognizing how they can make their models. With these modifications the models are much easier and cheaper for us to manufacture and for the students to build.

The designs for each of the plates, a detailed lesson plan, and guide for students are available as supplementary material.

### Cohort and teaching environment

The modeling tasks were carried out in a workshop session during the first semester of the first year of undergraduate study as part of a unit (module) entitled ‘Introduction to Neuroscience’. Whilst compulsory for students studying for a Neuroscience B.Sc., the unit is also open for other students to take, including undergraduates studying for a B.Sc. in other science subjects. All students follow the same program of study within the unit.

The study was conducted over three years with three separate cohorts totaling 308 students. The cohort was made up predominantly of students studying Neuroscience (~65% of students) with the remainder studying Physiological Science, Pharmacology or Applied Anatomy. Prior to the workshop, lectures had been delivered on the anatomy and function of three spinal cord tracts: the Anterolateral/Spinothalamic tract (ALS), the Dorsal-Column Medial Lemniscus tract (DCML) and the Corticospinal tract (CST). Lectures were recorded and made available to all students, allowing those who did not attend the lectures access to the same information. Students had previously attended neuroanatomy classes in the human dissection rooms where they had been able to handle prosected specimens of the spinal cord, brainstem and cerebrum. During this time, they also had access to eBioLabs (www.bris.ac.uk/ebiolabs/about), our virtual laboratory manual, which provided information on all three spinal cord tracts.

Three modeling stations were set out on eight separate benches in a large teaching laboratory. Students were asked to sit in groups (maximum of 4) at one of the three stations set out on each bench and were randomly assigned one of the three tracts to build. Thus, students did not have a choice of which tract to make.

### Pre-modeling research

Prior to beginning modeling, students were asked to spend 30 minutes researching all three individual tracts using whatever materials they wished to use. This included all the teaching material we had used through the unit (lecture notes/slides and textbooks). In addition, we used the 3D Human Brain Model (Zygote, 2015) as the basis for making 3D computer models of the spinal tracts. High resolution polygon models of the ascending sensory and descending motor tracts were constructed using Autodesk^®^ 3D Studio Max, which were used to generate bespoke digital resources for students to use alongside their anatomy classes. These were:

1. Interactive animations with captions but no commentary (not publicly available, hosted on eBioLabs).
2. YouTube videos with a character driven commentary, (publicly available, hosted at the Bristol Neuroscientists channel: https://www.youtube.com/@bristolneuroscientists1094).
3. Manipulatable 3D model (publicly available, hosted at http://www.sketchfab.com/anajd).

### Model building

Students were provided with the Simplified BrainTowers and colored pipe cleaners. Their initial task was to build the Simplified BrainTower itself. A colored guide was provided for them to check their work and to ensure the plates were placed in the right order. Holes cut in the acrylic plates represented the pathways for different spinal cord tracts. This part of the activity meant that students had to build a 3D representation of the brain and spinal cord from scratch.

Following the construction of the Simplified BrainTower, students then used colored pipe cleaners to represent the tract they had been assigned to model. Students were encouraged to be creative in their use of the pipe cleaners but to also be accurate in their representation. They were given up to 1 hour to complete the building of the model using the notes produced earlier and any further research they wished to conduct. For each model there were five structural features that needed to be shown:

1. number of neurons in the pathway and the location of each synapse
2. correct route of the pathway
3. entry/exit points in the spinal cord associated with upper and lower limbs
4. decussation points
5. cortical origin/target

During both building phases, tower and pathway, members of staff (2 lecturers and 2 demonstrators) were on hand to check the models and advise students if any mistakes had been made. However, students were not told specifically what the mistakes were, requiring further research and adjustments. After an hour of building, the models were checked again for mistakes. Students were then asked to describe their model to the two other groups on their bench. They were asked to point out the positions of the cortical origins/targets in relation to whether the pathway represented the upper or lower limbs (upper limbs should be on the lateral surface while lower limbs should be represented on the medial surface), the decussation points, entry/exit points and the number of neurons in the pathway. A representative fully-constructed model is shown in Fig 1D.

### Data Collection and analysis

Following the modeling, students were asked to put away their notes and turn off the computer screens. They were then asked questions about each of the tracts using a Turning Point quiz (now renamed Point Solutions; Stowell & Nelson, 2007). This formative quiz contained three questions about each of the tracts (ALS, CST and DCML), based on the features that had to be included in the model and the students’ own descriptions to their peers. A question to identify which tract each student had built was also included. Responses were collected and the data sorted into groups based on which model the student had built. Each student’s scores for answers to questions about each tract were collated. The quiz was repeated 5 days later to assess whether any improvements in performance had lasted over that period.

As part of the workshop, students had to prepare a piece of summative written work (a figure legend) with an anonymous tracking number that made it possible to determine which responses on the final assessments corresponded to the spinal cord tracts that had been built without identifying individual students. Thus, data from the final summative anatomy spot examination and end-of-unit examination were also collected. The spot exam consisted of a timed exam containing 25 specimens that the student had to identify and then answer an open response question about each structure, but which was not dependent on correct identification. One question about each of the three spinal cord tracts was included in the exam. The end-of-unit exam also included questions related to each of the three tracts. Answers were indicated on an Optical Mark Reading sheet (OMR) and automatically marked. All marking was conducted electronically and checked by the Unit Director.

To control for variations in the difficulty of any individual question, all quantitative data is expressed as a Performance Ratio (PR). The mean score for each individual student in one modeling group was compared to the average score on the same question(s) for all students in the other two modeling groups. This gives a ratio value which can indicate the relative performance of the two groups of students in answering the same questions. For example, for students who had built the ALS model, their individual mean scores were compared to the mean scores for the same questions from all students who had built the CST and DCML tract models. A PR value of 1 would indicate no improvement in performance for one group of students over the other. The performance ratios for questions related to the tract built by the modeling group were then compared to questions related to the other two tracts (related vs unrelated questions). A higher PR value for questions related to the modeled tract over unrelated questions would indicate a better learning outcome from the hands-on activity. All results are expressed as mean ± SEM. Statistical analysis was conducted using SPSS (IBM Corp), using two-tailed, paired T-tests, with an alpha value of 0.05. A Bonferroni correction has been applied to account for multiple hypotheses testing such that a result is considered significant at a p value <0.0167.

### Ethical considerations

At the time of data collection for this study (2016-2019), we were advised by the University of Bristol Research Ethics Committee that ethics approval was not required for evaluation of teaching sessions as data from the whole cohort was to be utilized and no individual student could be identified from the processed results. Students were informed about how we would evaluate the outcomes for the workshop and could withdraw permission to use data for this purpose – no student withdrew permission over the three years.

## RESULTS

### Building a model of a spinal cord tract enhances students’ knowledge of that tract

**Figure 2.**
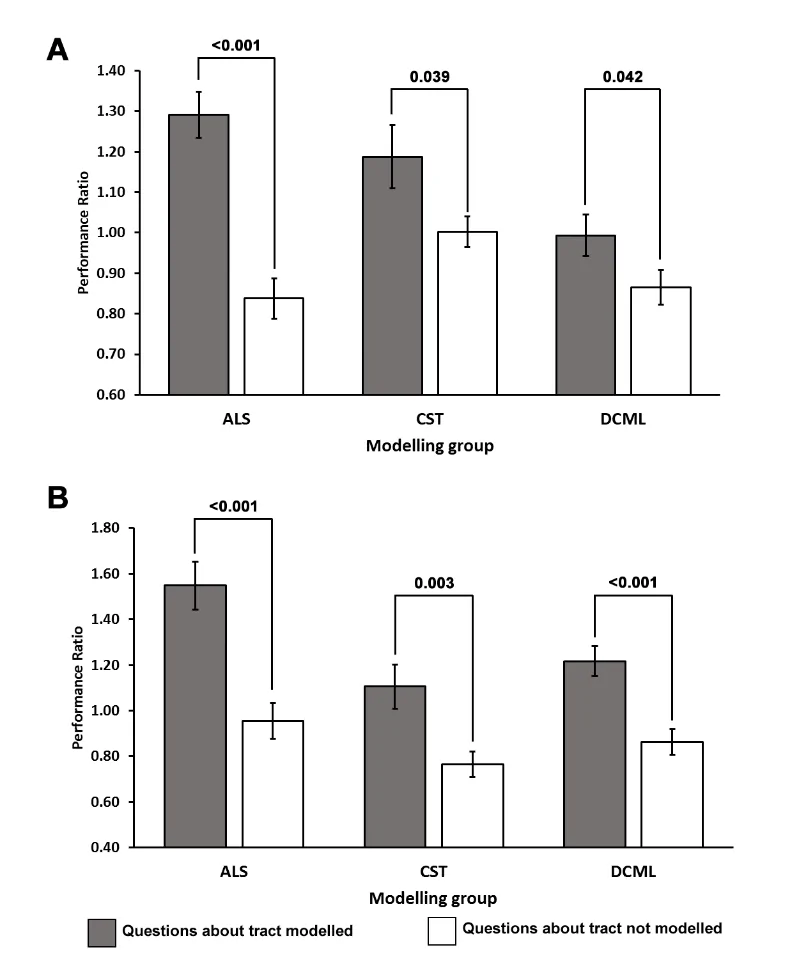
Building a model improves test scores on questions relating to that model. First year students were asked questions in a Turning Point quiz relating to all three tested spinal cord tracts: ALS – anterolateral/spinothalamic tract, CST – corticospinal tracts, DCML – dorsal column-medial lemniscus tract. Data is represented as the performance ratio (mean ± SEM) for questions related to the tract that was modeled (related question topic) and questions related to the tracts that were not modeled (unrelated question topic). Questions related to the modeled topic were answered more effectively by students who built the model than questions relating to the non-modeled tracts. p values for each test are noted, two-tailed, paired T-test. Panel A - questions asked immediately after the activity (n=189). Panel B – questions asked 5 days after the activity (n=85).

One of the key aims of this study was to determine if model building enhanced knowledge of the spinal cord tracts. This was tested using a turning point quiz that tested students’ knowledge without reference to any learning materials after modeling one tract. The results are shown in Figure 2. Data is presented as the performance ratios for related and unrelated questions within each modeling group. Immediately following the modeling activity, students performed better in the quiz on questions related to the model they made versus unrelated questions (Fig 2A). Thus, students in the ALS modeling group displayed a significant increase in performance ratio on questions related to the ALS tract compared to those related to the CST/DCML tracts with a mean difference in PR value of 0.45 (95% CI [0.33,0.57]). Smaller differences were seen for both the CST modeling group, with mean difference in PR values for related vs unrelated questions of 0.18 (95% CI [0.01, 0.36]) and the DCML modeling group, with a mean difference in PR value of 0.13 (95% CI [0.01, 0.26]). Thus, there appears to be an immediate increase in students’ knowledge of the basic anatomy of the tracts they modeled during the session, with the clearest change in the ALS modeling group.

To test whether this enhancement in knowledge was retained, the same questions asked in the workshop were repeated to students in a tutorial 5 days after the initial workshop. Timetabling changes during the third year of data collection meant that it was not possible to hold the follow-up tutorial. Thus, the data presented represents the student responses from the first two years only. Students were not aware that these questions were going to asked again and so did not specifically prepare for the test. The results are shown in Figure 2B. There were clear, significant improvements in the test scores from students who modeled the ALS and DCML tracts. For those modeling the ALS tract, there was a mean difference in PR value of 0.59 (95% CI [0.38, 0.81]). This pattern was repeated for the other modeling groups, with mean differences in PR score of 0.34 (95% CI [0.12, 0.56]) and 0.35 (95% CI [0.20, 0.51]) for those modeling the CST and DCML tracts, respectively.

### Model building enhances assessment outcome scores.

The next question we wanted to answer is whether the modeling activity improved the scores under exam conditions. Students undertake two relevant summative assessments during the first year of study – the anatomy ‘spot’ exam and the end-of-unit exam. Summative assessments were carefully prepared to ensure that no group of students would be disadvantaged, with questions about each of the tracts included. Thus, if there were to be any positive effect on scores, each modeling group would be advantaged equally.

**Figure 3.**
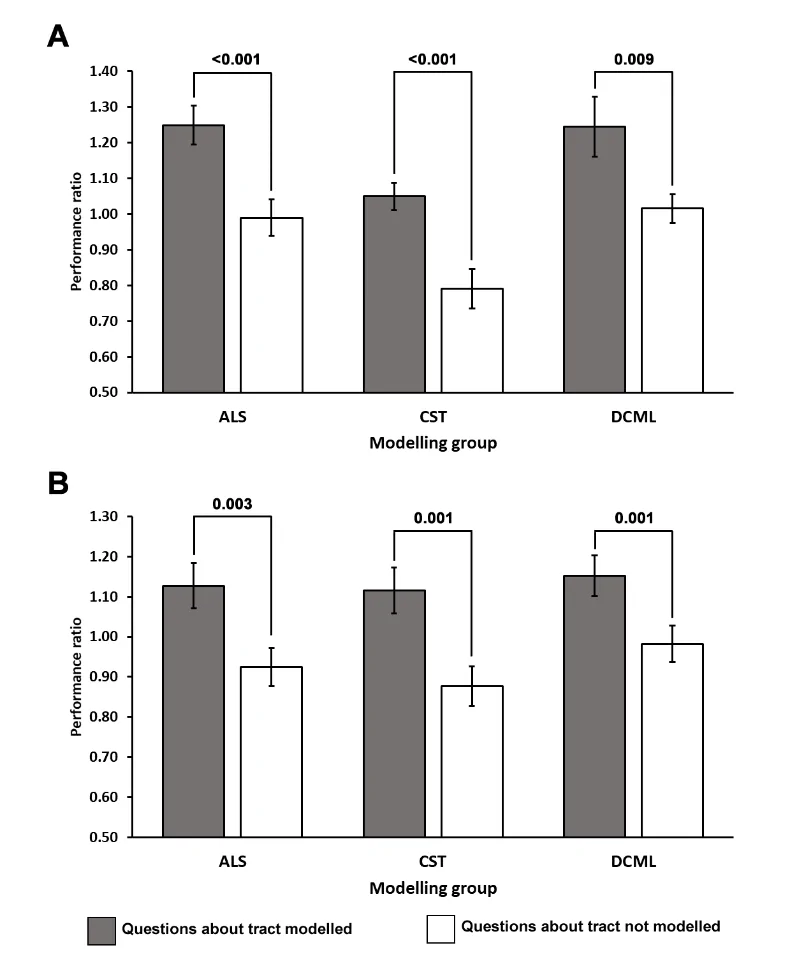
Improvement in test scores from model building is evident in summative examinations. Students sat two summative examinations during the unit - a neuroanatomy spot exam 5 weeks after the workshop activity (Panel A, n=185) and the end-of-unit exam (Panel B, n=186), 11 weeks after the workshop activity. Data is represented as in Figure 1. Each group of students answered questions related to their modeled tract more effectively than questions related to the non-modeled tracts. p values for each test are noted, two-tailed, paired T-test. ALS – anterolateral/spinothalamic tract, CST – corticospinal tracts, DCML – dorsal column-medial lemniscus tract.

*Anatomy spot exam:* Students took a neuroanatomy spot exam 5 weeks after the initial modeling activity. The exam consists of 25 questions, covering a wide range of different anatomical specimens. A single question related to each tract was included in this assessment. The PR scores for students in each of the modeling groups are shown in Figure 3A. There is an apparent improvement in PR values for students answering the questions aligned to the modeling groups versus those that were not. The mean difference in PR values for the related vs unrelated questions were: 0.26 (95% CI [0.12,0.38]) for the ALS group, 0.26 (95% CI [0.14, 0.38]) for the CST group and 0.22 (95% CI [0.06, 0.40]) for the DCML group.

*End-of-Unit Exam:* Students sat an end-of-unit exam 11 weeks after the workshop and modeling activity. The exam consisted of 80 questions related to all aspects of teaching on the unit. Questions related to each of the three tracts that had been modeled were included. The PR scores for students in each of the modeling groups are shown in Figure 3B. There is an improvement in PR values for students answering the questions aligned to the modeling groups versus those that were not. The mean difference in PR values for the related vs unrelated questions were: 0.20 (95% CI [0.07,0.33]) for the ALS group, 0.24 (95% CI [0.10, 0.38]) for the CST group and 0.17 (95% CI [0.07, 0.26]) for the DCML group.

These results show that not only does the modeling help with the initial acquisition of knowledge, but that such knowledge can be retained, recalled, and applied many weeks later.

### Student perception of activity

**Figure 4.**
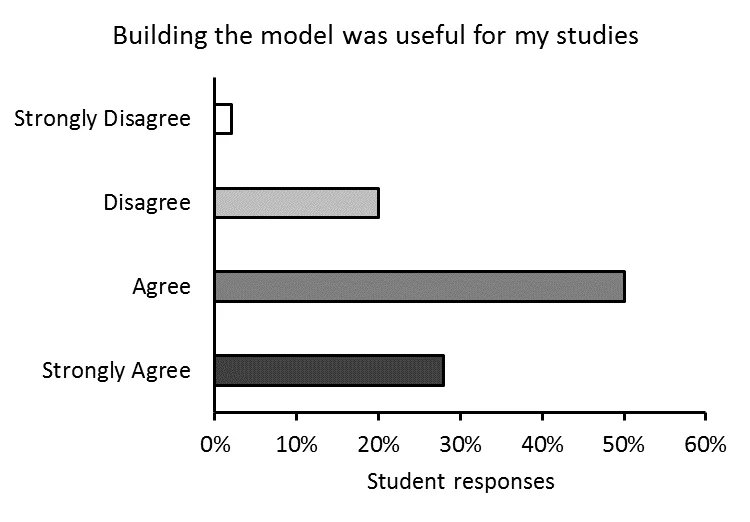
Student perception of modeling activity. Students were asked to agree or disagree with the statement ‘Building the model was useful for my studies’. Responses were collected via Turning Point clickers (n=50).

Students were asked to rate the usefulness of the workshop during the end-of-unit feedback session via Turning Point^®^ questions. When asked about the modeling activity, 78% of students who responded agreed or strongly agreed with the proposition that building the model was useful for their studies (Figure 4).

## DISCUSSION

One of the key aims of this study was to determine whether teaching activities that include model building are useful in improving students’ knowledge of anatomical structures. The data clearly show that model building enhances student knowledge of the structure they build. Students modeling any one of the spinal cord tracts could answer questions about that tract better than questions related to the tracts they did not model. An enhancement in performance is seen immediately after the activity and importantly, the information is retained into the near future (Fig 2). The performance enhancements were also maintained into the final summative assessments (Fig 3). Interestingly, the questions used in the summative anatomy spot exam tested related information rather than the direct anatomy. Thus, these results suggest that model building enables students to not only gain better basic anatomical knowledge but can also help integrate related information to synthesize a greater understanding of the role of those tracts in biological systems.

A major question that arises from this data is whether the enhancements in performance can be attributed to the modeling activity, the prior research carried out by students or the subsequent summative writing task. We can rule out an effect of the prior research as all students were asked to research all three tracts before being assigned a tract to build. However, it is difficult to separate the effects of the modeling activity itself from the subsequent writing task. The time spent writing the figure legend will certainly be expected to have an impact on students’ knowledge retention of the tract they had built. Indeed, using multiple modalities for teaching is strongly aligned to enhanced learning and retention of information (Sugand et al., 2010). It is possible to see effect of the writing exercise in the increase in the PR difference score for all modeling groups 5 days after the workshop; this is the period when students complete their written summative assessment, based on the tract they modeled.

The impact of the modeling itself on learning can be demonstrated through the Turning Point^®^ quiz during the workshop (Fig 2A). In addition, it should be noted that students have many opportunities to revise the anatomy of each tract, but they only make the model once. Indeed, we encourage our students to engage with equivalent writing tasks using drawings of the other tracts as part of their revision. We expect that this would dilute the effect of the summative task. In this context, it is interesting that students building the ALS model demonstrated enhanced learning over the students building the DCML and CST tracts when tested within 5 days of the modeling exercise (Fig 2). It is not clear why this was the case, but may be due to the relatively straightforward anatomy of the ALS pathway compared to the others; students building this tract often finish first. However, by the time the summative assessments are taken some weeks later, the apparent advantage of modeling the ALS tract was lost (Fig 3). Indeed, the enhancement in learning for all the tracts was lower but stable at these assessments, supporting the notion that further revision would dilute the effect of the modeling activity, but that it is still present, suggesting that the modeling itself has a significant impact on learning. Further work is necessary to determine the relative impact of physically interacting with the model and time spent on other related tasks.

There are other questions that need to be considered in using models for teaching. Models can be expensive to purchase and unless students actively interact with them, they may be no better than didactic lectures as a learning tool (Preece et al., 2013). On the other hand, active learning processes have been shown to be very beneficial for student learning (Hyun et al., 2017; Michael, 2006) and these tasks can take many forms. Actively building or deconstructing a model brings many benefits in terms of the learning achieved. For instance, clay modeling has been shown to be at least as effective in teaching students anatomy as live dissection (DeHoff et al., 2011; Motoike et al., 2009) and more effective than didactic approaches (Akle et al., 2017; Estevez et al., 2010; Oh et al., 2009). Similarly, using modeling clay to build representations of ion channels and neurons has been shown to be beneficial to students’ critical thinking skills (Krontiris-Litowitz, 2003) while using paper models has been shown to improve students’ knowledge of the musculoskeletal system (Rehorek et al., 2019). Drawing is another active learning technique that has been shown to improve learning outcomes (Na et al., 2022; Quillin & Thomas, 2015). It can be used not only to assess students’ knowledge (Slominski et al., 2017) but also to engage students in constructing and synthesizing their own knowledge (Ainsworth et al., 2011). In common with our iteration of the BrainTower^®^, these activities are simple to prepare, and students intuitively know what to do to complete the tasks.

In this context, it is very interesting to note how the students perceived the usefulness of this activity. At the end-of-unit evaluation, students were asked to rate the model building activity using Turning Point^®^ clickers. Nearly 80% of students who responded reported that they found it a useful activity. Whilst relatively small numbers of students took part in the end-of-unit feedback, from the chatter and laughter in the classes, as well as personal conversations with individual students, the activity seemed to be enjoyable and engaging. From the question scores, students clearly learnt and retained relevant information across several weeks, although students were unaware of this at the time. Previous studies have also shown that students appreciate the educational value in building anatomical models (Arya et al., 2013; Correia et al., 2022). This stands in stark contrast to the perceived usefulness of the original model, where 77% of students disagreed that the BrainTower^®^ was useful in their studies (Davies et al., 2014). This exemplifies the need to make active learning tasks simple and intuitive in order to engage students in the process and so improve learning.

The data derived in this study demonstrates that the use of such a simple and intuitive activity is very effective at engaging students and so enabling learning. It also suggests that deep rather than surface learning is achieved by students building a model themselves (Marton & Saljo, 1976) rather than through other means. The improvements in students’ ability to answer questions on related topics were not only maintained for at least eleven weeks, but questions about the function of the modeled tract were also answered better than the other tracts, suggesting a higher level of learning than simply recall of information (Bloom et al., 1956). In this regard, it is interesting to note that critical thinking skills have been shown to be enhanced by making clay models of ion channels (Krontiris-Litowitz, 2003) although it should be noted that active learning also improves students’ ability to simply remember facts (Cherney, 2008). In addition, a recent study showed that using physical course materials for formative work enabled greater understanding of structure-function relationships (Forbes-Lorman et al., 2022). Thus, building models has multiple benefits in allowing students to explore not only how a structure is put together, but also in how it functions, two critical areas of learning that are essential in the study of functional anatomy.

Finally, we observed long-lasting differences between modeling groups that depended on the tract students modeled. This has implications for how we use active learning methodologies and in particular the design of subsequent summative assessments. It would be very easy to set summative work where one group of students may be advantaged over another. Thus, we were very careful to ensure that all summative assessments contained questions on all three tracts, so as not to advantage or disadvantage any groups of students. In an ideal world, students should build all three models and so gain insights into the anatomy of all three tracts. Timetabling and time constraints make this unrealistic. However, with careful consideration of how the models are used and the assessment criteria, the Simplified BrainTower is a powerful active learning tool that will help many students gain a better understanding of the anatomy and function of the ascending and descending tracts.

In conclusion, we have demonstrated that building models of anatomical structures is a very useful tool, especially when teaching elements of anatomy that are difficult to show on cadaveric specimens. Not only does this type of activity improve students’ learning of the structures being modeled, but it allows them to build a scaffold on which to hang other related information, inducing a deep form of learning that should hopefully last, long after the activity itself has been forgotten.

### Address correspondence to

Dr. Andrew Doherty, School of Physiology, Pharmacology and Neuroscience, University of Bristol, Biomedical Sciences Building, University Walk, Bristol, UK, BS8 1TD.

Email: a.doherty@bristol.ac.uk

Copyright © 2025 Faculty for Undergraduate Neuroscience

www.funjournal.org

## Supplementary Material

Supplementary Material 1 Designs for each of the plates, a detailed lesson plan, and guide for students.

Supplementary Material 2 BrainTower Questions
