# Supplementary material for: The Simplified BrainTower and Pipe Cleaners: Model Building as a Learning Tool in Neuroscience
Source: J Undergrad Neurosci Educ. 2025 Dec 31;24(1):27–37. doi: 10.59390/001c.153902 (PMC13127669; doi:10.59390/001c.153902)
Supplement: Supplementary Material 1 — Designs for each of the plates, a detailed lesson plan, and guide for students. [file junejournal_2025_24_1_153902_320789.zip › Simplified Brain Towers - plates and glossary.docx]

Use the images below to decide on the order in which the Simplified Brain Tower should be built.


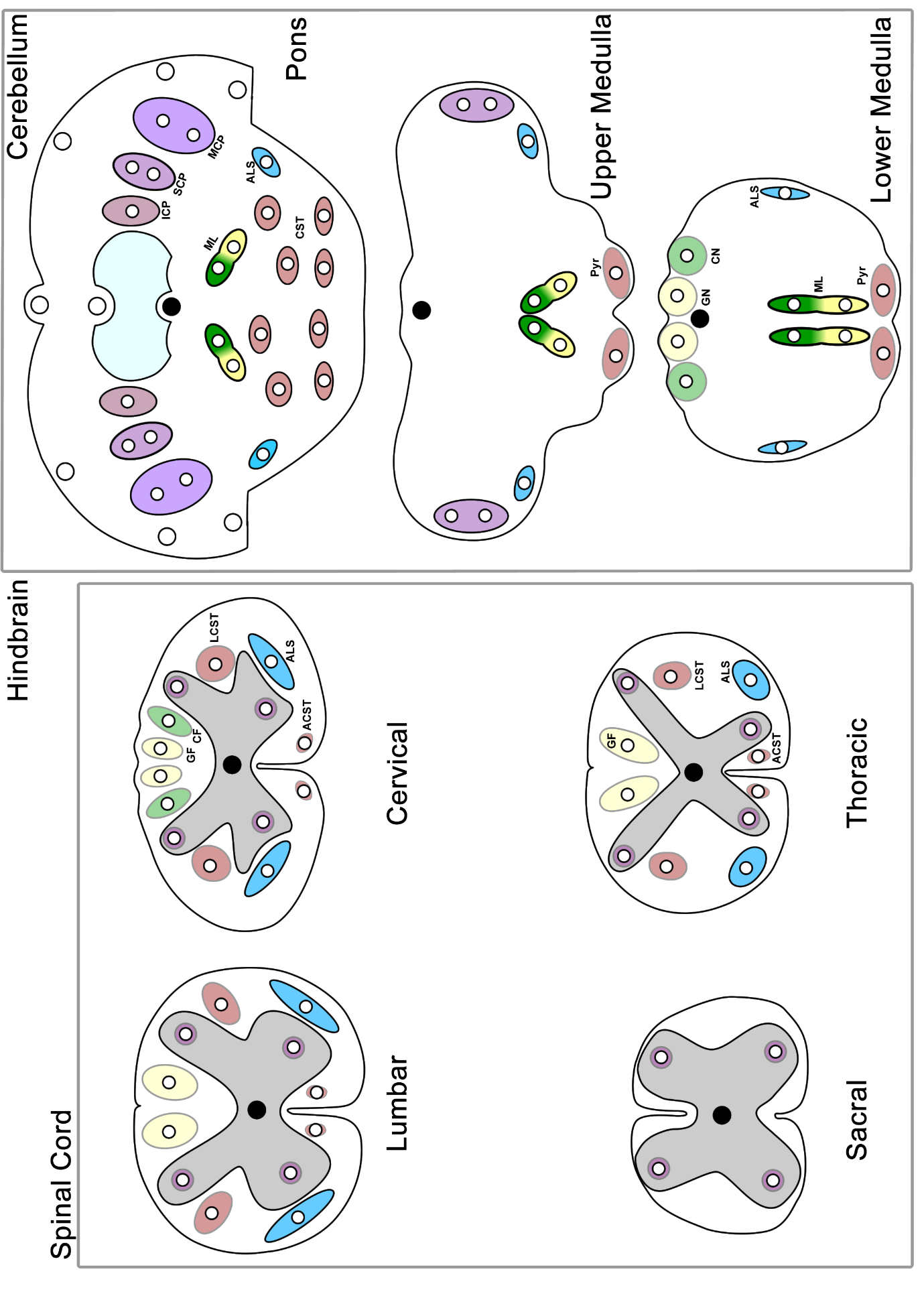


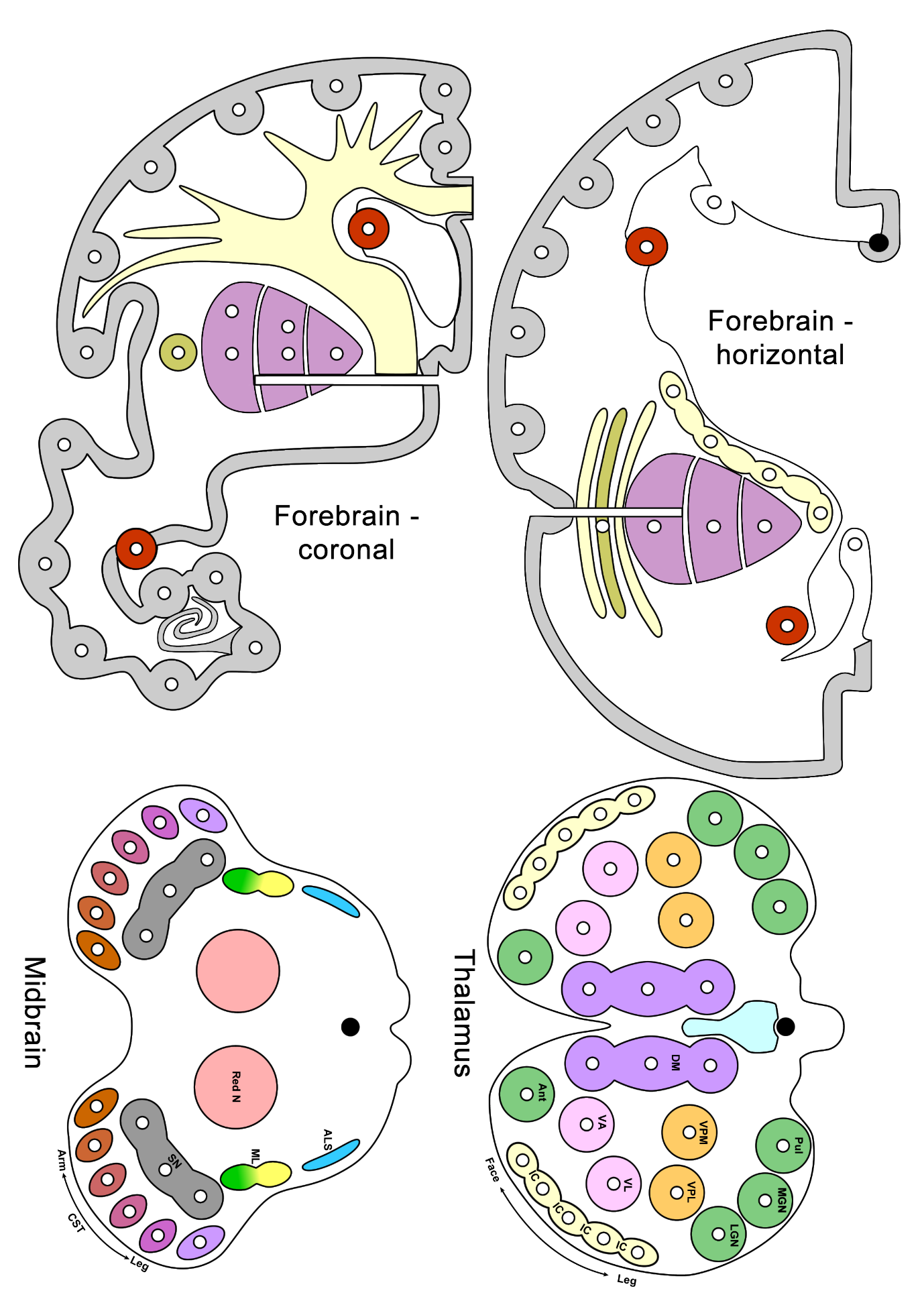


Glossary

ACST – Anterior corticospinal tract

Ant – Anterior nucleus of the thalamus

ALS – Anterolateral System

CF – Cuneate fasciculus

CN – Cuneate nucleus

CST – Corticospinal tract

DM – Dorsomedial nucleus of the thalamus

GF – Gracile fasciculus

GN – Gracile nucleus

IC – Internal capsule

ICP – Inferior cerebellar peduncle

LCST – Lateral corticospinal tract

LGN – Lateral geniculate nucleus

MGN – Medial geniculate nucleus

ML – Medial lemniscus

MCP – Middle cerebellar peduncle

Pul - Pulvinar

Pyr – Pyramid

Red N – Red nucleus

SCP – Superior cerebellar peduncle

SN – Substantia nigra

VA – Ventroanterior nucleus of the thalamus

VL - Ventrolateral nucleus of the thalamus

VPL - Ventroposterolateral nucleus of the thalamus

VPM - Ventroposteromedial nucleus of the thalamus
