# Supplementary material for: The Simplified BrainTower and Pipe Cleaners: Model Building as a Learning Tool in Neuroscience
Source: J Undergrad Neurosci Educ. 2025 Dec 31;24(1):27–37. doi: 10.59390/001c.153902 (PMC13127669; doi:10.59390/001c.153902)
Supplement: Supplementary Material 1 — Designs for each of the plates, a detailed lesson plan, and guide for students. [file junejournal_2025_24_1_153902_320789.zip › Simplified Brain Towers - Workshop Plan.docx]

**Simplified Brain Towers – Workshop plan**

The workshop runs over the course of 3 hours and is aimed at understanding the overall anatomy of sensory and motor tracts within the CNS. Students work in groups of 3-4 and we use this activity during the first semester of their first year of study.

Three specific tracts are used to illustrate the organisation of these tracts – the Dorsal Colum Medial Lemniscus Tracts (DCML), Anterolateral Spinothalamic Tract (ALS) and the Corticospinal Tract (CST). The DCML and ALS are ascending sensory tracts while the CST is a descending motor tract. While students build only one model, they are given time to compare their model with the others being made and can note the differences and similarities between them.

The workshop is divided into sections devoted to knowledge acquisition, model building and presentation of findings.

**Section 1. Research (30 mins)**

Students should spend some time looking at the structure and anatomy of these three tracts. They should have had teaching previously about all the relevant tracts and should be encouraged to use their lecture notes as well as information available online to familiarise themselves with each tract. At this stage, students do not know which tract they are going to build. Questions about the three tracts can be asked at the end of the research phase.

**Section 2. Modelling (60 mins)**

- ***Build the Simplified Brain Tower®***

The students are first asked to build the Simplified Brain Tower. They are provided with the relevant materials and a guide showing the different sections that are used to build the model (*Simplified Brain Towers – plates and glossary*). Each section is named and the tracts colour coded. Students can use these images to decide on the order in which the sections are placed. A glossary of terms is also provided as is an image of the sensory or motor homunculus, preferably one that has been used in previous teaching. Staff should check the order of the sections prior to building the tract to ensure that mistakes are rectified (Figure 1).

- ***Build the relevant tract***

Students are assigned a tract to build. They can use the colour coded guide or any other information they find useful to construct the pathway of the tract using coloured pipe-cleaners. The pathway should include five specific elements:

1. Innervation to/from the ARMS and LEGS
2. Routes through the spinal cord, brain stem and cortex
3. Points of decussation i.e. where do the tracts cross the midline
4. Correct number of neurons for the tract being represented
5. Location of cortical territory

Encourage students to think about how the different neurons in the tract can be represented. For example, 1^st^, 2^nd^ and 3^rd^ order neurons of the sensory tracts from the ARMS could be represented by different variants of ‘hot’ colours (reds, pinks, oranges) while the same neurons form the LEGS could be represented by ‘cool’ colours (blues, purples, greens). That way the pathways between the cortical territories and peripheral location can be easily seen. We also encourage the students to be as creative as they wish to be and they often embellish their models with representations of, for instance, hands and feet to demonstrate their understanding of the somatotopy of the spinal cord.

Logistically, it is good to ensure that group building the different tracts are located near each other for the next part of the workshop.

**Section 3. Sharing (30 mins)**

Students should spend 5 mins each describing their models to groups that made the other 2 tracts. In this way they can compare the structure of each, reflect on the differences between them and think about the consequences for function.

**Section 4. Questions (20 mins)**

Questions related to each of the tracts can be asked in 2 phases – after the research phase and after sharing phase. Ensure that there are the same number of questions for each tract and include a question asking which tract was built.

Finally, students should take a photo of their model – we use this as part of their written summative assessment for the unit as a whole but could simply be a reminder of a fun day with pipe-cleaners!

**Supporting teaching**

This workshop is supported by other workshops and tutorials held during semester 1, in particular two tutorials on the structure of the spinal cord and the effects of lesions (e.g. via a stroke) in different parts of the CNS. Students must use their knowledge of these tracts to explain the functional losses induced by the lesion. This enables them to link the anatomy of the tracts to their function and how the loss of function can be used to identify the location of a lesion.

**Other uses for the Simplified Brain Tower**

The Simplified Brain Tower can be used to represent any number of other tracts in the CNS. We have used it to illustrate the Trigeminal tracts and it can easily be used to model the different pathways of the basal nuclei (basal ganglia). Addition of the deep cerebellar nuclei to the relevant section would enable the modelling of the cerebellar network.

**Note:** we now run this workshop over the course of 2 hours rather than 3 with timings as follows:

**Introduction** – 10 mins

**Research** - 15 mins

**Modelling** (both tower and tract) – 50-60 mins

**Sharing** – 15 mins

The questions section is missed out, leaving enough time for students to get into the room and get settled before starting that task.


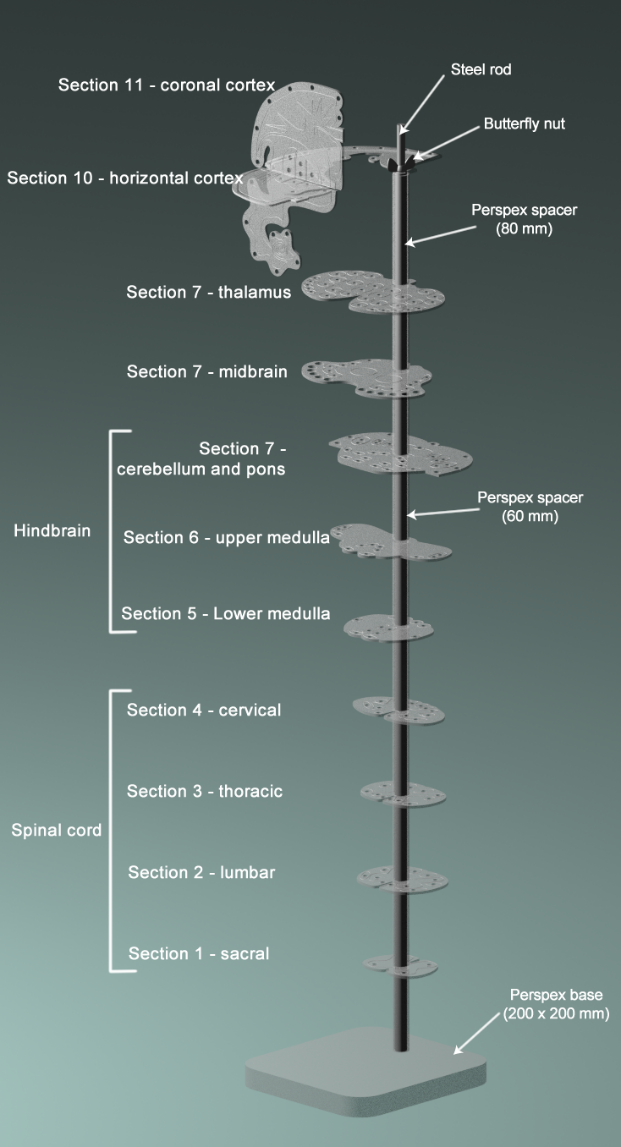


Figure 1. Completed Simplified Brain Tower®.

Use the images below to decide on the order in which the Simplified Brain Tower® should be built.


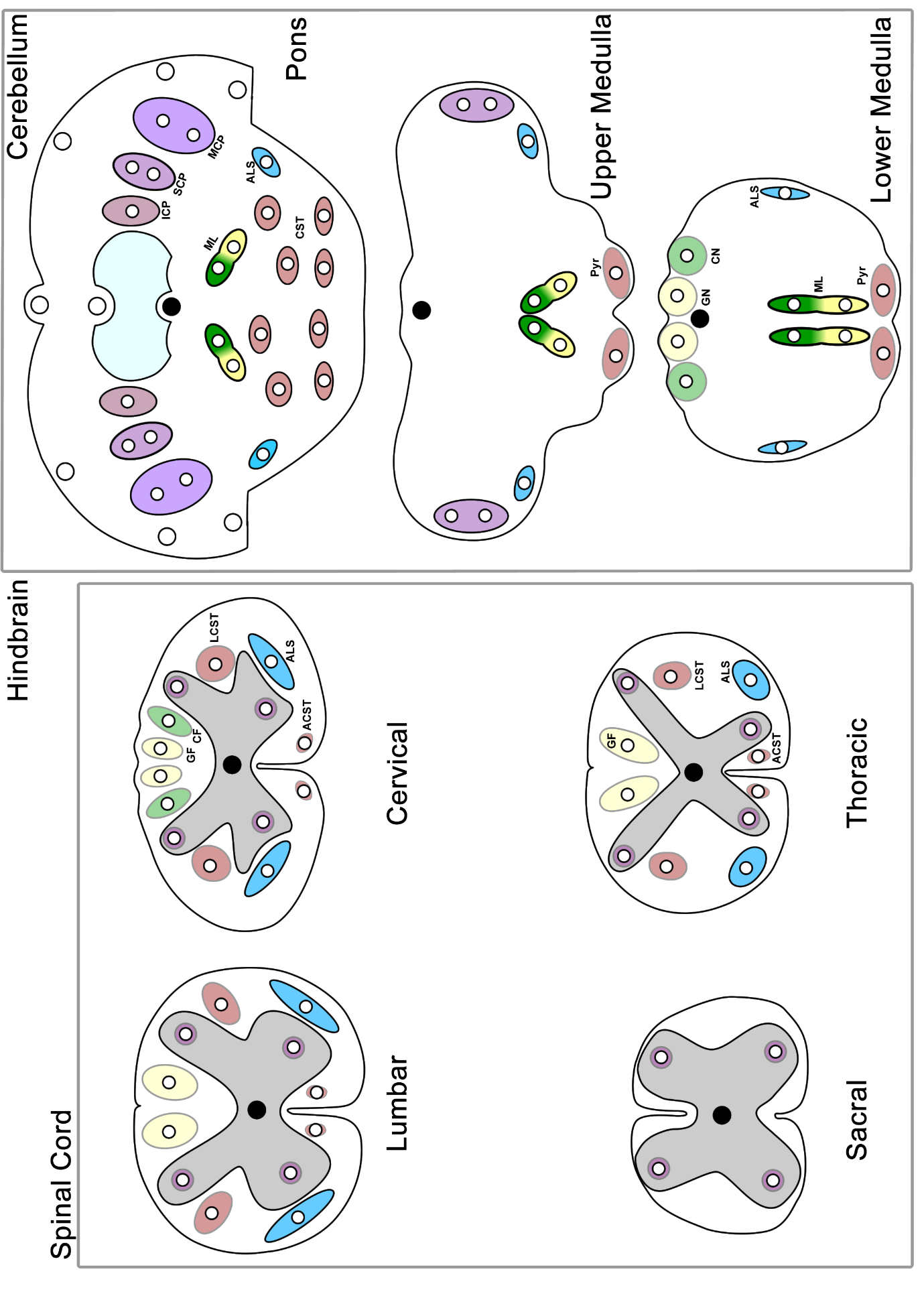


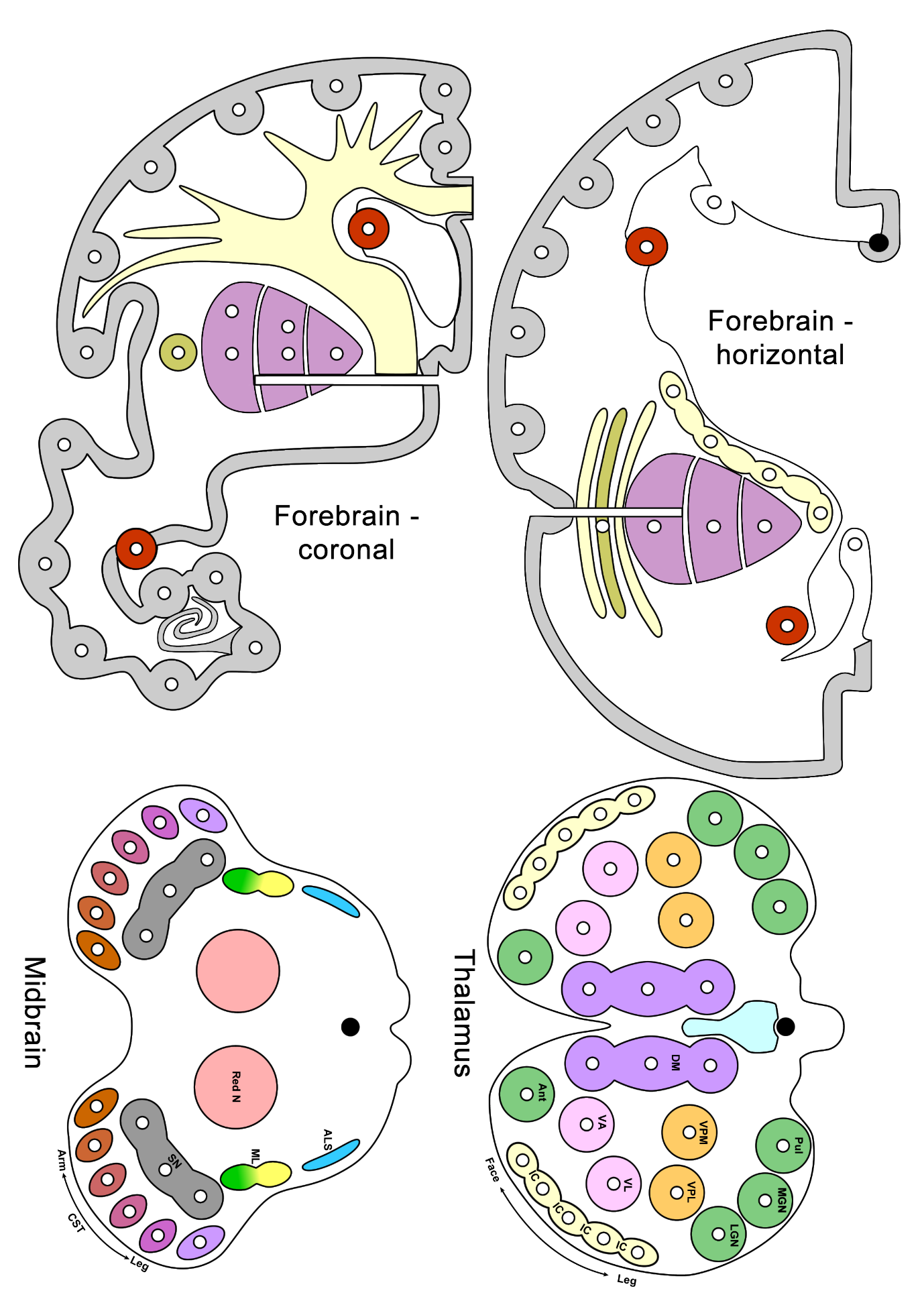


Glossary

ACST – Anterior corticospinal tract

Ant – Anterior nucleus of the thalamus

ALS – Anterolateral System

CF – Cuneate fasciculus

CN – Cuneate nucleus

CST – Corticospinal tract

DM – Dorsomedial nucleus of the thalamus

GF – Gracile fasciculus

GN – Gracile nucleus

IC – Internal capsule

ICP – Inferior cerebellar peduncle

LCST – Lateral corticospinal tract

LGN – Lateral geniculate nucleus

MGN – Medial geniculate nucleus

ML – Medial lemniscus

MCP – Middle cerebellar peduncle

Pul - Pulvinar

Pyr – Pyramid

Red N – Red nucleus

SCP – Superior cerebellar peduncle

SN – Substantia nigra

VA – Ventroanterior nucleus of the thalamus

VL - Ventrolateral nucleus of the thalamus

VPL - Ventroposterolateral nucleus of the thalamus

VPM - Ventroposteromedial nucleus of the thalamus
