# Supplementary material for: The Simplified BrainTower and Pipe Cleaners: Model Building as a Learning Tool in Neuroscience
Source: J Undergrad Neurosci Educ. 2025 Dec 31;24(1):27–37. doi: 10.59390/001c.153902 (PMC13127669; doi:10.59390/001c.153902)
Supplement: Supplementary Material 2 — BrainTower Questions [file junejournal_2025_24_1_153902_320790.docx]

Questions on tracts: There were 9 questions asked in the workshop related to the three tracts and one to identify which tract was built.

1. Which tract did you make?
   1. Dorsal column medial lemniscus
   2. Anterolateral/spinothalamic
   3. Corticospinal
2. How many neurons are there in the corticospinal tract?
   1. 1
   2. 2
   3. 3
   4. 4
   5. 5
3. Where does the corticospinal tract decussate?
   1. Rostral/cranial end of the spinal cord
   2. At entry in the spinal cord
   3. Caudal end of the spinal cord
   4. Below the pyramids in the medulla
   5. Rostral/cranial end of the medulla
   6. At the crus cerebri in the midbrain
4. How many neurons are there in the dorsal column tract?
   1. 1
   2. 2
   3. 3
   4. 4
   5. 5
5. Where does the anterolateral tract decussate?
   1. Rostral/cranial end of the spinal cord
   2. At entry in the spinal cord
   3. Caudal end of the spinal cord
   4. Below the pyramids in the medulla
   5. Rostral/cranial end of the medulla
   6. At the crus cerebri in the midbrain
6. At which nucleus of the thalamus does the dorsal column tract synapse?
   1. Ventrolateral (VL)
   2. Ventroposteromedial (VPM)
   3. Ventroanterior (VA)
   4. Ventromedial (VM)
   5. Ventroposterolateral (VPL)
7.
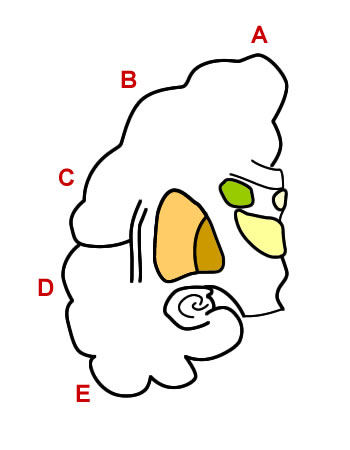
In which part of the somatosensory cortex would a pain in the foot be registered?
   1. A
   2. B
   3. C
   4. D
   5. E
8. Where does the dorsal column tract decussate?
   1. Rostral/cranial end of the spinal cord
   2. At entry in the spinal cord
   3. Caudal end of the spinal cord
   4. Below the pyramids in the medulla
   5. Rostral/cranial end of the medulla
   6. At the crus cerebri in the midbrain
9.
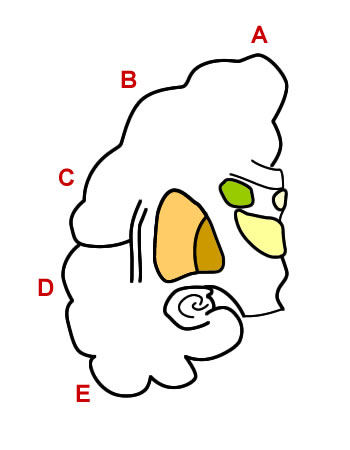
Which of these statements is true? Choose up to 3 answers.
   1. Neurons from **D** project through the internal capsule as part of the corticospinal tract
   2. Region **B** detects pressure information from the hand via the DCML pathway
   3. Region **E** receives pain information from face via the anterolateral system
   4. Control of muscles in the face arise from region **C**
   5. Region **A** receives pain information from leg via the anterolateral system
10. How many neurons are there in the anterolateral/spinothalamic tract?
    1. 1
    2. 2
    3. 3
    4. 4
    5. 5
